# Preferred analysis methods for Affymetrix GeneChips revealed by a wholly defined control dataset

**DOI:** 10.1186/gb-2005-6-2-r16

**Published:** 2005-01-28

**Authors:** Sung E Choe, Michael Boutros, Alan M Michelson, George M Church, Marc S Halfon

**Affiliations:** 1 Department of Genetics, Harvard Medical School, New Research Building, 77 Avenue Louis Pasteur, Boston, MA 02115, USA; 2 Division of Genetics, Department of Medicine, Brigham and Women's Hospital, New Research Building, 77 Avenue Louis Pasteur, Boston, MA 02115, USA; 3 Howard Hughes Medical Institute, Brigham and Women's Hospital, 20 Shattuck Street, Boston, MA 02115, USA; 4 Department of Biochemistry, 140 Farber Hall, 3435 Main St., SUNY at Buffalo, Buffalo, NY 14214, USA; 5 Center of Excellence in Bioinformatics, 140 Farber Hall, 3435 Main St., SUNY at Buffalo, Buffalo, NY 14214, USA; 6 German Cancer Research Center (DKFZ/B110), Im Neuenheimer Feld 580, 69120 Heidelberg, Germany

## Abstract

**Background:**

As more methods are developed to analyze RNA-profiling data, assessing their performance using control datasets becomes increasingly important.

**Results:**

We present a 'spike-in' experiment for Affymetrix GeneChips that provides a defined dataset of 3,860 RNA species, which we use to evaluate analysis options for identifying differentially expressed genes. The experimental design incorporates two novel features. First, to obtain accurate estimates of false-positive and false-negative rates, 100-200 RNAs are spiked in at each fold-change level of interest, ranging from 1.2 to 4-fold. Second, instead of using an uncharacterized background RNA sample, a set of 2,551 RNA species is used as the constant (1x) set, allowing us to know whether any given probe set is truly present or absent. Application of a large number of analysis methods to this dataset reveals clear variation in their ability to identify differentially expressed genes. False-negative and false-positive rates are minimized when the following options are chosen: subtracting nonspecific signal from the PM probe intensities; performing an intensity-dependent normalization at the probe set level; and incorporating a signal intensity-dependent standard deviation in the test statistic.

**Conclusions:**

A best-route combination of analysis methods is presented that allows detection of approximately 70% of true positives before reaching a 10% false-discovery rate. We highlight areas in need of improvement, including better estimate of false-discovery rates and decreased false-negative rates.

## Abstract 2

A 'spike-in' experiment for Affymetrix GeneChips is described that provides a defined dataset of 3,860 RNA species. A 'best route' combination of analysis methods is presented which allows detection of approximately 70% of true positives before reaching a 10% false discovery rate.

## Background

Since their introduction in the mid 1990s [1,2], expression-profiling methods have become a widespread tool in numerous areas of biological and biomedical research. However, choosing a method for analyzing microarray data is a daunting task. Dozens of methods have been proposed for the analysis of both high-density oligonucleotide (for example, Affymetrix GeneChip) and spotted cDNA or long oligonucleotide arrays, with more being put forward on a regular basis [3]. Moreover, it is clear that different methods can produce substantially different results. For example, two lists of differentially expressed genes generated from the same dataset can display as little as 60-70% overlap when analyzed using different methods ([4] and see Additional data file 1). Despite the large number of proposed algorithms, there are relatively few studies that assess their relative performance [5-9]. A significant challenge to undertaking such studies is the scarcity of control datasets that contain a sufficiently large number of known differentially expressed genes to obtain adequate statistics. The comparative studies that have been performed have used a small number of positive controls, and have included a background RNA sample in which the concentrations of the various genes are unknown, preventing an accurate assessment of false-positive rates and nonspecific hybridization.

The most useful control datasets to date for evaluating the effectiveness of analysis methods for Affymetrix arrays are cRNA spike-in datasets from Affymetrix and Gene Logic. The Affymetrix Latin square dataset [10] is a series of transcriptional profiles of the same biological RNA sample, into which 42 cRNAs have been spiked at various known concentrations. The dataset is designed so that, when comparing any two hybridizations in the series, all known fold changes are powers of two. The Gene Logic dataset [11] has a similar experimental design, but with 11 cRNAs spiked in at varying fold changes, ranging from 1.3-fold upwards.

Here we present a new control dataset for the purpose of evaluating methods for identifying differentially expressed genes (DEGs) between two sets of replicated hybridizations to Affymetrix GeneChips. This dataset has several features to facilitate the relative assessment of different analysis options. First, rather than containing a limited number of spiked-in cRNAs, the current dataset has 1309 individual cRNAs that differ by known relative concentrations between the spike-in and control samples. This large number of defined RNAs enables us to generate accurate estimates of false-negative and false-positive rates at each fold-change level. Second, the dataset includes low fold changes, beginning at only a 1.2-fold concentration difference. This is important, as small fold changes can be biologically relevant, yet are frequently overlooked in microarray datasets because of a lack of knowledge as to how reliably such small changes can be detected. Third, our dataset uses a defined background sample of 2,551 RNA species present at identical concentrations in both sets of microarrays, rather than a biological RNA sample of unknown composition. This background RNA population is sufficiently large for normalization purposes, yet also enables us to observe the distribution of truly nonspecific signal from probe sets which correspond to RNAs not present in the sample.

We have used this dataset to compare several algorithms commonly used for microarray analysis. To perform a direct comparison of the selected methods at each stage of analysis, we applied all possible combinations of options to the data. Thus, it was possible to assess whether some steps are more critical than others in maximizing the detection of true DEGs. Our results show that at several steps of analysis, large differences exist in the effectiveness of the various options that we considered. These key steps are: first, adjusting the perfect match probe signal with an estimate of nonspecific signal (the method from MAS 5.0 [12] performs best); second, checking that the log fold changes are roughly distributed around 0 (by observing the so-called M versus A plot [13], the plot of log fold change (M) *versus *average log signal intensity (A)), and if necessary, performing a normalization at the probe-set level to center this plot around M = 0; and third, choosing the best test statistic (the regularized *t*-statistic from CyberT [14] is most accurate). Overall, we find a significant limit to the sensitivity of microarray experiments to detect small changes: in the best-case scenario we could detect approximately 95% of true DEGs with changes greater than twofold, but less than 30% with changes below 1.7-fold before exceeding a 10% false-discovery rate. We propose a 'best-route' combination of existing methods to achieve the most accurate assessment of DEGs in Affymetrix experiments.

## Results and discussion

### Experimental design

**Figure 1:**
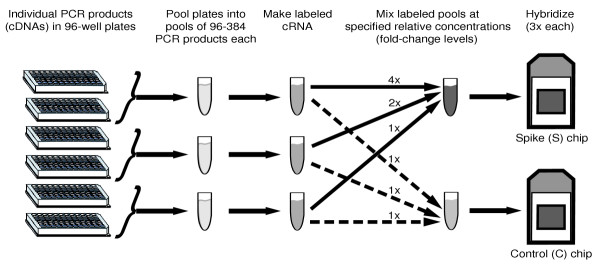
Schematic depiction of the experimental protocol.

A common use of microarrays is to compare two samples, for example, a treatment and a control, to identify genes that are differentially expressed. We constructed a control dataset to mimic this scenario using 3,860 individual cRNAs of known sequence in a concentration range similar to what would be used in an actual experimental situation (see Materials and methods). The cRNAs were divided into two samples - 'constant' (C) and 'spike' (S) - and each sample was hybridized in triplicate to Affymetrix GeneChips (six chips total). The S sample contains the same cRNAs as the C sample, except that selected groups of approximately 180 cRNAs each are present at a defined increased concentration compared to the C sample (Figure 1, Table 1). Out of the 3,860 cRNAs, 1,309 were spiked in with differing concentrations between the S and C samples. The rest (2,551) are present at identical relative concentration in each sample, to serve as a guide for normalization between the two sets of microarrays. For the sake of consistency with typical discussions of microarray experiments, we sometimes refer to the cRNAs with positive log fold changes as DEGs, despite their not representing true gene-expression data.

### Assignment of Affymetrix probe sets to DGC clones

In the Affymetrix GeneChip design, the expression level of each RNA species is reported by a probe set, which in the DrosGenome1 chip [15] comprises 14 oligonucleotide probe pairs. Each probe pair contains two 25-mer DNA oligonucleotide probes; the perfect match (or PM) probe matches perfectly to the target RNA, and the mismatch (or MM) probe is identical to its PM partner probe except for a single homomeric mismatch at the central base-pair position, and thus serves to estimate nonspecific signal.

The DrosGenome1 chip used in this experiment is based on release version 1.0 of the *Drosophila *genome sequence and thus does not represent the most up-to-date annotated version of the genome. To ensure that probe-target assignments are made correctly, we assigned the 14,010 probe sets on the DrosGenome1 GeneChip to the spiked-in RNAs by BLAST of the individual PM probe sequences against the *Drosophila *Gene Collection release 1.0 (DGC [16]) clone sequences that served as the template for the cRNA samples (Materials and methods). Of the 3,860 DGC clones used in this study, 3,762 (97%) have full-length cDNA sequence available at the DGC web site, 90 have 3' and 5'-end sequence only, and eight have no available sequence. For each probe set, all clone sequences with BLAST matches to PM probe sequences in that probe set are collected, allowing at most two (out of 25 base-pair (bp)) mismatches, and only allowing matches on the correct strand. If at least three PM sequences match to a given clone, then the probe set is assigned to that clone. Matches of one probe set to more than one clone are allowed. In this manner, 3,866 probe sets are assigned to at least one DGC clone each. Among these probe sets, 1,331 have an increased concentration between the S and C chips, whereas 2,535 represent RNAs with equal concentration between the two samples. Among those probe sets which do not have any assignment using this criterion, if fewer than three PM probes within the probe set have a BLAST match to any clone, the probe set is then called 'empty' (that is, its signal should correspond to nonspecific hybridization). There are 10,131 empty probe sets; combined with the 2,535 1x probe sets, about 90% of the probe sets on the chip represent RNAs with constant expression level between the C and S samples. The rest of the probe sets are then called 'mixed', meaning that they match to more than one clone, but each with only a few PM probe matches. There are only 13 mixed probe sets. The numbers of probe sets assigned to each fold-change class are depicted in Table 1.

### Assessment of absent/present call metrics

Our dataset design provides the rare knowledge of virtually all of the RNA sequences within a complex sample (excepting the small number (3%) of clones for which only partial sequence was available, and the possible rare mistakenly assigned or contaminated clone). We can therefore evaluate various absent/present call metrics on the basis of their ability to distinguish between the known present and absent RNAs. We investigate this issue at both the probe pair level and probe set level. For the probe pair level assessment, we first identify the probe pairs which we expect to show signal, and those which should not. We thus define two classes of probe pairs: first, perfect probe pairs, whose PM probe matches perfectly to a target RNA sequence, and neither PM nor MM probe matches to any other RNA in the sample with a BLAST E-value cutoff of 1 and word size of 7, and second, empty probe pairs, whose PM and MM probes do not match to any RNA sequence when using the same criteria.

**Figure 2:**
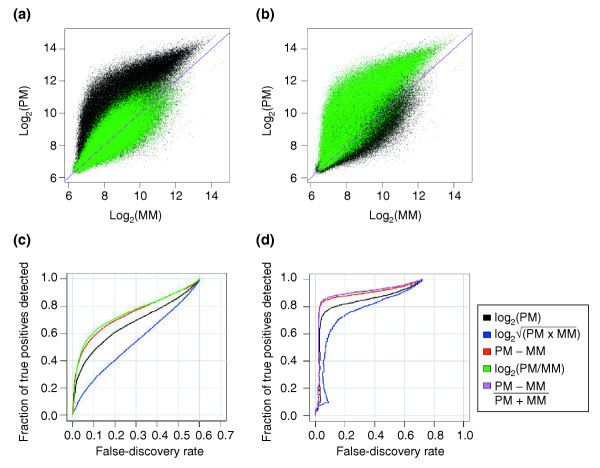
Signal of individual probes and dependence on present versus absent RNA molecules. **(a, b) **Plot of probe-pair signals for the three C chips, highlighting (a) the empty probe pairs or (b) the present probe pairs in green. **(c) **Receiver-operator characteristic (ROC) curves at the probe-pair level for several absent/present metrics. The metric (PM - MM)/(PM + MM) gives the same result as the green curve. **(d)**Receiver-operator characteristic curves at the probe-set level for several absent/present metrics combined using the Wilcoxon rank sum test.

On the chip, which contains 195,994 probe pairs, there are 50,859 perfect probe pairs and 117,904 empty ones. Observation of the signal for these probe pairs (Figure 2a,b) clearly shows that there is considerable signal intensity for the empty probe pairs. Figure 2c shows the ability of several metrics - log_2_(PM/MM), PM-MM, , and log_2_(PM) - to distinguish between perfect and empty probe pairs, by calculating receiver-operator characteristics (ROC) curves using the perfect probe pairs as true positives and the empty ones as true negatives. Each point on a curve depicts the specificity and sensitivity for RNA detection, when using a specific value of the corresponding metric as a cutoff for classifying probe sets as present or absent. Instead of depicting the false-positive rate (the fraction of true negatives that are detected as present) on the *x*-axis, which is customary for these types of graphs, we show the false-discovery rate (the fraction of detected probe sets which are true negatives), which distinguishes between the metrics more effectively for the top-scoring probe sets. Figure 2 clearly shows that metrics that compare the PM signal with the MM signal, such as log_2_(PM/MM) and PM-MM, are the most successful at distinguishing perfect from empty probe pairs. This indicates that the PM signal alone is a less effective indicator of RNA presence, probably because the probe hybridization affinity is highly sequence-dependent. However, even with the more successful metrics, only about 60% of the perfect probe sets are detected before reaching a 10% false-discovery rate, indicating that there is still a high level of variability in probe pair sensitivity, even when using the MM signal to estimate the probe hybridization affinity.

When signals from the 14 probe pairs in each probe set are combined to create a composite absence/presence call, a much larger fraction of the spiked-in RNA species can be detected reliably. To obtain absent/present calls at the probe-set level, we perform the Wilcoxon signed rank test using each of the metrics listed above [17]. The *p*-values from this test are used to generate the ROC curves in Figure 2d. Again, the best results are obtained when the metric compares PM with MM signals, as opposed to monitoring signal alone. The metric used in MAS 5.0 ((PM-MM)/(PM+MM)), which is equivalent to log_2_(PM/MM), performs best. Therefore, the MM signals are important in generating accurate presence/absence calls. In our dataset, about 85% of the true positives could be detected before having a 10% false-discovery rate. The detection of perfect probe pairs is not improved when we include additional information from replicates. The 15% of probe sets which are called absent may represent truly absent RNAs, owing to failed transcription or labeling (see Additional data file 5). However, as we do not have an independent measure of failed transcription for the individual cRNA sequences in the target sample, we cannot completely rule out the possibility that they are the result of non-responsive probes or a suboptimal absent/present metric that fails to score low-abundance cRNAs. Regardless, as non-responsive probes or missing target cRNAs should affect both the C and S chips identically, these factors should not limit the value of this dataset in making relative assessments of different analysis methods.

### Generating expression summary values

**Figure 3:**
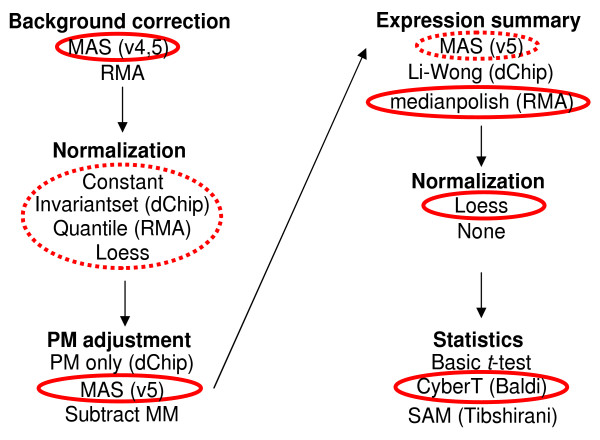
The set of options that were investigated using Bioconductor's affy package. The choices that optimize the detection of DEGs are circled in red. Broken circles indicate choices that are slightly suboptimal but still rank within the top 10 datasets.

The first task in analyzing Affymetrix microarrays is to combine the 14 PM and 14 MM probe intensities into a single number ('expression summary') which reflects the concentration of the probe set's target RNA species. Generating this value involves several discrete steps designed to subtract background levels, normalize signal intensities between arrays and correct for nonspecific hybridization. To compare the effectiveness of different analysis packages at each of these steps, we created multiple expression summary datasets using every possible (that is, compatible) combination of the options described below. Algorithms were chosen for their popularity with microarray researchers and their open-source availability, and were generated using the implementations found in the Bioconductor 'affy' package [18]. Figure 3 summarizes the options that we chose within Bioconductor. We also used the dChip [19] and MAS 5.0 [12] executables made available by the respective authors in order to cross-check with the open-source implementations within Bioconductor. In addition, we applied two analysis methods that incorporate probe sequence-dependent models of nonspecific signal (*Perfect Match *[20] and *gcrma *[21]). The combinations of options that were used to generate the 152 expression summary datasets are detailed in Additional data file 2.

#### Background correction

An estimate of the background signal, which is the signal due to nonspecific binding of fluorescent molecules or the autofluorescence of the chip surface, was generated using two possible metrics. The *MAS *background [17] is calculated on the basis of the 2nd percentile signal in each of 16 subsections of the chip, and is thus a spatially varying metric. The Robust Multi-chip Average (*RMA*) algorithm [22] subtracts a background value which is based on modeling the PM signal intensities as a convolution of an exponential distribution of signal and a normal distribution of nonspecific signal.

#### Normalization at the probe level

The signal intensities are normalized between chips to allow comparisons between them. Because in our dataset, a large number of RNAs are increased in S versus C (and none are decreased), commonly used methods often result in apparent downregulation for spiked-in probe sets in the 1x change category. We thus added a set of modified normalization methods which used our knowledge of the 1x probe sets. The following different methods were applied. *Constant *is a global adjustment by a constant value to equalize the chip-wide mean (or median) signal intensity between chips. *Constantsubset *is the same global adjustment but equalizing the mean intensity for only the probe sets with fold change equal to 1. *Invariantset *[23] is a nonlinear, intensity-dependent normalization based on a subset of probes which have similar ranks (the rank-invariant set) between two chips. *Invariantsetsubset *is the same as invariantset but the rank-invariant set is selected as a subset of the probe sets with fold change equal to 1. *Loess *normalization [24] is a nonlinear intensity-dependent normalization which uses a local regression to make the median fold change equal to zero, at all average intensity levels. *Loesssubset *normalization is the same as loess but using only the probe sets with fold change equal to 1. *Quantile *normalization [24] enforces all the chips in a dataset to have the same distribution of signal intensity. *Quantilesubset *normalization is the same as quantile but normalizes the spiked-in and non-spiked-in probe sets separately.

#### PM correction

We chose three ways to adjust the PM signal intensities to account for nonspecific signal. The first is to subtract the corresponding MM probe signal (*subtractmm*). The second is the method used in *MAS *5.0, in which negative values are avoided by estimating the nonspecific signal when the MM value exceeds its corresponding PM intensity [17]. The third is *PM only *(no correction). The subtractmm and MAS methods are compatible only with the MAS background correction method; that is, it does not make sense to combine these with RMA background correction.

#### Expression summary

The 14 probe intensity values were combined using one of the following robust estimators: Tukey-biweight (*MAS *5.0); *median polish *(RMA); or the model-based *Li-Wong *expression index (dChip). Analyses including the *subtractmm *PM correction method require dealing with negative values when PM is less than MM, which occurs in about a third of the cases. Within Bioconductor, the *Li-Wong *estimator can handle negative values, but the other two metrics mostly output 'not applicable' (NA) for the probe set when any of the constituent probe pairs has negative PM - MM. The result for *MAS *and *median polish *is NA for about 85% of the probe sets on the chip. To study the consequence of losing so many probe sets, we modified one of these two metrics (*median polish*) to accept negative (PM - MM) (*medianpolishna*), and added this metric whenever *subtractmm *was used.

#### Normalization at the probe set level

**Figure 4:**
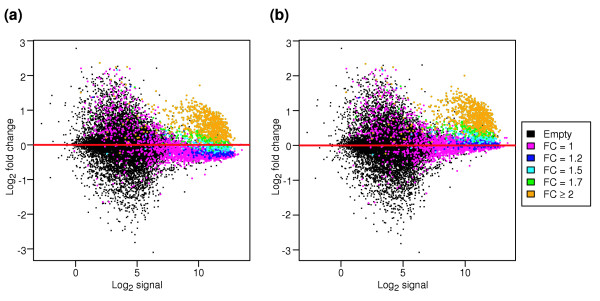
The dependence of log fold change on signal intensity (M versus A plots). **(a)**M versus A plot before the second normalization step and **(b) **after a loess fit at the probe set level. FC in the key denotes the spiked-in fold change value.

Many of the expression summary datasets that were produced still show a dependence of fold change on the signal intensity (Figure 4a). To correct this, a second set of expression summary datasets was created, in which a loess normalization at the probe set level was used to center the log-fold changes around zero (Figure 4b).

### Comparison of the observed fold changes with known fold changes

**Figure 5:**
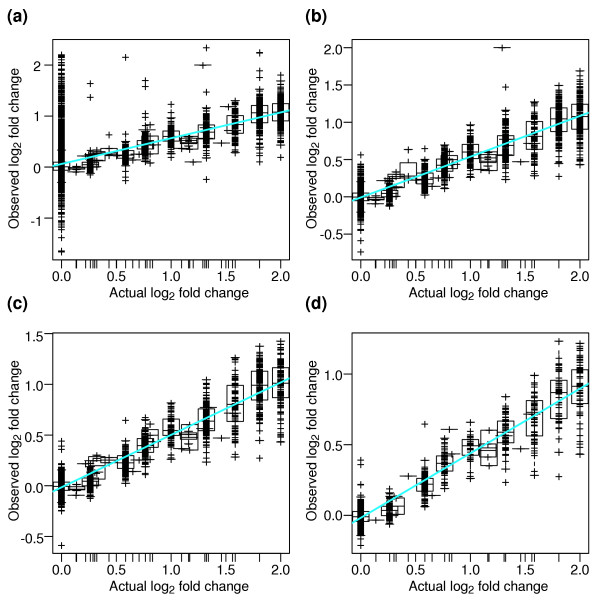
Correlation of observed with actual fold changes for a representative expression summary dataset (Additional data file 2, using dataset 9e.b). **(a) **The fold change for each probe set with spiked-in target RNA is depicted as a cross. Empty probe sets are not shown. For each actual fold-change level (on the *x *axis), a boxplot shows the distribution of the corresponding observed fold changes. A linear fit of the data is shown in cyan. Fit parameters: R^2 ^= 0.508; slope = 0.505; y-intercept = -0.061. **(b-d) **Increasingly more of the low-intensity probe sets are filtered out of the plot. All probe sets are ranked according to average signal level, and those in the lowest 25th (b), 50th (c), or 75th (d) percentile of signal level are eliminated from (a). Fit parameters: (b) R^2 ^= 0.870; slope = 0.546; y-intercept = -0.008; (c) R^2 ^= 0.895; slope = 0.517; y-intercept = -0.015; (d) R^2 ^= 0.906; slope = 0.457; y-intercept = -0.017.

For each of the 150 expression summary datasets that we generated, fold changes between the S and C samples were calculated and then compared with the actual fold changes. Most expression summary datasets show good correlation between the observed and actual fold changes (Figure 5). The greatest sources of variability are probe sets with low signal intensity; as Figure 5b shows, the correlation improves dramatically when we filter out the probe sets with low signal. For all the expression summary datasets, the agreement between observed and actual fold changes is good (R^2 ^= 86 ± 3%) when the probe sets in the lowest quartile of signal intensity are filtered out. The expression summary datasets which involve correcting the PM signal by subtracting the MM signal (*subtractmm*) have the highest correlation coefficient, because low-intensity probe sets have been filtered out during processing, as described above. We therefore suggest that an important feature of a successful microarray analysis is to account for probe sets with low signal intensity, either by filtering them out or by using a signal-dependent metric for significance. Several ways of accomplishing such filtering are described below.

We also observed that the fold changes resulting from the chips are consistently lower than the actual fold changes. Apparently, the decrease in fold change is only partly the result of signal saturation (Figure 5b-c), and is not a byproduct of the robust estimators used to calculate expression summaries (because the low fold changes are also observed at the probe pair level; see Additional data file 3). In other experiments we have also observed that our Affymetrix fold-change levels are smaller than those obtained by quantitative reverse transcription (RT)-PCR (data not shown). One likely explanation is that we do not have an adequate estimate for nonspecific signal. For example, if we choose the MM signal as the nonspecific signal (thus calculating PM - MM, or PM - CT from MAS 5.0), we are probably overestimating the nonspecific signal, as the MM intensity value responds to increasing target RNA concentrations, and therefore contains some real signal. On the other hand, if we choose not to use a probe sequence-dependent nonspecific signal (such as in RMA), we are likely to underestimate the nonspecific signal for a large number of probes. In either case, the result is decreased fold change magnitudes. Artificially low fold-change values have been noted by others, including those investigating the Affymetrix Latin square [6], GeneLogic [22] and other [25] datasets, although some of the differences they report are smaller than are observed here.

### Test statistics and ROC curves

**Figure 6:**
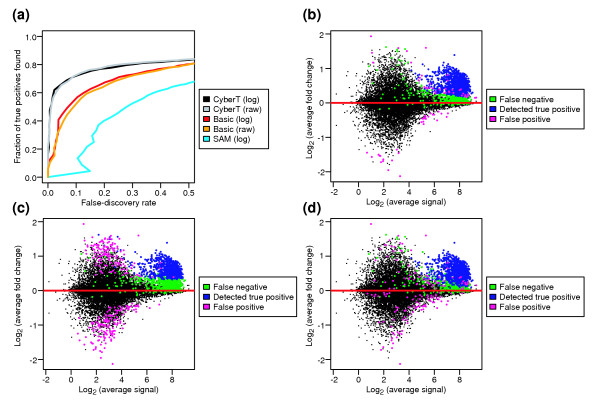
Comparison of three *t*-statistic variants. **(a)**ROC curves for a particular expression summary dataset, using the different *t*-statistics. Location of false positives and false negatives are shown for the **(b) **CyberT, **(c) **SAM, and **(d) **basic *t*-statistic when considering the top 1,000 probe sets as positive DEG calls.

Because a typical microarray experiment contains a large number of hypotheses (here 14,010) and a limited number of replicates (in this case three), high false-positive rates are a common problem in identifying DEGs. An important factor in minimizing false positives is to incorporate an appropriate error model into the signal/noise metric. We compared three *t*-statistic variants, which differ in their calculations of noise. The first is significance analysis for microarrays (*SAM*) [26], in which the *t*-statistic has a constant value added to the standard deviation. This constant 'fudge factor' is chosen to minimize the dependence of the *t*-statistic variance on standard deviation levels. The second is *CyberT *[14], in which the standard deviation is modeled as a function of signal intensity. The third is the *basic *(Student's) *t*-statistic. For *CyberT *and the *basic t*-test, we performed the tests on the expression summaries after log transformation, as well as on the raw data. As shown in the example ROC curve, the *CyberT *statistic outperforms the other statistics for the vast majority of expression summary datasets (Figure 6a). Inspection of the false positives and false negatives shows the reason for the different performance. Because *CyberT *uses a signal intensity-dependent standard deviation, probe sets at low signal intensities have reduced significance even when their observed fold change is high (Figure 6b). As shown in Figure 6c, the *SAM *algorithm (using the authors' Excel Add-in) does not effectively filter out these same false-positive probe sets (with low signal intensity and high fold change). Upon further inspection, we observed that the *SAM *algorithm favors using large values for the constant fudge factor, so that the *t*-statistic depends more on the fold change value, than on the noise level. The *basic t*-statistic is prone to false positives resulting from artificially low standard deviations, owing to the limited number of replicates in a typical microarray experiment (scattered magenta spots in Figure 6d). This comparison agrees with the result of Broberg [9], who also found that the *CyberT *approach (there called 'samroc') outperforms several other methods. Because the *CyberT *statistic clearly performs the best, we use only this statistic to compare the options for the other steps in microarray analysis, below.

### Comparison of options at each of the other analysis steps

**Figure 7:**
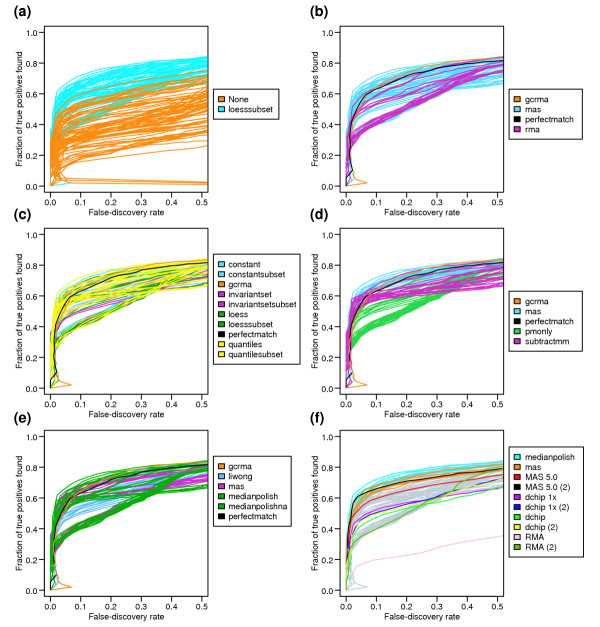
ROC curves for all expression summary datasets. The curves are color-coded to highlight how the ability to detect differential expression is dependent on the different options at each step of analysis, using the CyberT regularized *t*-statistic metric. **(a) **All 152 expression summary datasets are represented here, with the different colors depicting whether the second loess normalization step at the probe set level was performed. In general, the second loess normalization (blue) improves the detection of true DEGs. **(b-f)**To decrease clutter, only the 76 expression summary datasets involving the second normalization step are shown. (b) When comparing the two background correction methods, the MAS algorithm is superior to the RMA algorithm. (c) The various probe-level normalization methods do not show great differences between each other. (d) Among the different PM-correction options, using the method in MAS 5.0 clearly is the most successful. (e) Various robust estimators were examined, revealing that the median polish method is the most sensitive (with MAS 5.0's Tukey Biweight a close second). (f) Depiction (in blue and orange) of the 10 datasets which maximize detection of truly differentially expressed genes, while minimizing false positives. These datasets are generated using the options circled in Figure 3. MAS 5.0, with the inclusion of the second loess normalization step, falls within these top 10.

Performance of the various options that were investigated varied significantly, as seen by the ROC curves shown in Figure 7. First, we find that a second loess normalization at the probe set level generally yields a superior result (Figure 7a,f), as could be expected by observing the strong intensity-dependence of the fold-change values in Figure 4. This intensity-dependence is most likely the result of the unequal concentrations of labeled cRNA for the C and S chips. However, this artifact is not unique to this dataset. We routinely observe similar intensity-dependent fold changes in comparisons of biological samples, especially when there are small differences in starting RNA amounts between the two samples (see Additional data file 4 for an example). Therefore, in the absence of a biological reason to suppose that the fold change should depend on signal intensity, it is important to view the plot of log fold change versus signal and recenter it around *y *= 0 when necessary. Owing to the significant improvement seen when the second normalization is used, the subsequent figures (Figure 7b-f) only show the comparison of the remaining options in conjunction with this step (blue curves in Figure 7a).

Among the background correction methods, the *MAS *5.0 method generally performs better than the *RMA *method (Figure 7b). No clearly superior normalization method was found at the probe level (Figure 7c), even when using the subset normalization variants, although quantile normalization tended to underperform in the absence of the second normalization step.

With respect to adjusting the PM probe intensity with an estimate of nonspecific signal, Figure 7d clearly shows that either subtracting the MM signal (*subtractmm*), or using the *MAS *5.0 correction method, is better than using uncorrected or RMA-corrected PM values (*PM-only*). The *MAS *5.0 method performs the best because it does not create any negative values. This result is in apparent conflict with the conclusions of Irizarry *et al*. [5], who show drastically reduced noise at low signal intensity levels when the PM signal is not adjusted with MM values, and therefore better detection of spiked-in probe sets when using the fold change as the cutoff criterion. However, when Irizarry *et al*. use a test statistic that takes the variance into account, *PM-only *and MM-corrected methods (*MAS*) have similar sensitivity/specificity (Figure 3d,e from [5]). In the dataset presented here, the *MAS *PM-correction method yields a high variance at low signal-intensity levels, which effectively reduces the false-positive calls at this intensity range when using CyberT, thus resulting in better performance than when using *PM-only*. We can reconcile the Irizarry *et al*. result with our observations by considering a major difference between the datasets used by the two studies. Both the Affymetrix and GeneLogic Latin square datasets used in [5] involve a small number (10-20) of spiked-in cRNAs in a common biological RNA sample, and therefore comparisons are made between two samples that are almost exactly the same. As a result, the nonspecific component of any given probe's signal is expected to be almost identical in the two samples, and should not contribute to false-positive differential expression calls. In contrast, a large fraction of our dataset is differentially expressed; in addition, the C sample contains a high concentration of (unlabeled) poly(C) RNA. Because nonspecific hybridization depends both on a probe's affinity and on the concentrations of RNAs that can hybridize to it in a nonspecific fashion, we expect that each probe's signal can have different contributions of nonspecific hybridization between the C and S chips. Figure 2a shows that nonspecific hybridization can be a large component of a probe's signal. We hypothesize that, for our dataset, *PM-only *performs worse than MM-corrected methods (*subtractmm *or *MAS*) because *PM-only *does not try to correct for nonspecific hybridization in a probe-specific fashion. In contrast, for the Latin square datasets used in [5], *PM-only *works just as well as MM-corrected methods because the contribution of nonspecific hybridization is constant. Therefore, datasets which compare substantially different RNA samples (such as two different tissue types) should probably be processed using the *MAS *5.0 method for PM correction.

Figure 7e compares the different robust estimators that were used to create expression summaries. Of these, *median polish *(RMA) and the Tukey Biweight methods (*MAS *5.0) perform the best. Figure 7f highlights the 10 best summary method option sets, which are also depicted in Figure 3, as well as straight applications of some popular software, with or without an additional normalization step at the probe-set level. The result from the MAS 5.0 software, when adjusted with the second loess normalization step, ranks among the top 10. However, the other methods (dChip, RMA and MAS 5.0 without probe-set normalization) are not as sensitive or specific at detecting DEGs.

We were concerned that some of our analyses might be confounded by a possible correlation between low fold change and low expression summary levels, which could affect the interpretations of Figure 7 (comparing different methods) and the detection of small fold changes (see below). We therefore examined the distribution of expression levels within each spiked-in fold change group, and compared the methods with respect to their ability to detect a subset of probe sets with low expression summary levels (Additional data file 5). We found that the distribution of expression levels for the known DEGs was comparable among all the fold-change groups, and that all the conclusions reported here are similarly applicable to the low expression subset. However, the sensitivity of all methods was reduced, suggesting that they perform less well on weakly expressed than on highly expressed genes. As the number of low signal spike-ins was relatively small (265 probe sets), resulting in reduced accuracy for the ROC curves, the development of additional control datasets specifically focusing on DEG detection at low cRNA concentrations will be an important extension of this study.

Models dependent on probe sequence provide a promising route to improving the accuracy of nonspecific signal measures. Here, we applied two different models (*perfect match *and *gcrma*) to the control dataset. With respect to detecting the true DEGs, these two models perform reasonably well, although slightly less well than the MAS 5.0 PM correction method. When we consider only the low signal DEGs (Additional data file 5), *gcrma *outperforms perfect match, and is similar in effectiveness to the top analysis option combinations.

### Estimating false discovery rates

**Figure 8:**
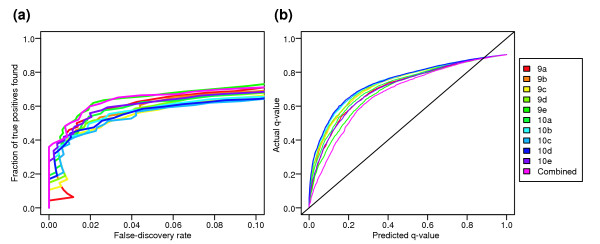
The accuracy of false discovery rate estimates (*q*-values). The top 10 expression summary datasets (named 9a-9e, 10a-10e in Additional data file 2) were combined to generate a composite statistic, which was used to rank genes based on the robustness of their significance over the 10 datasets. **(a) **The composite statistic performs as well as the best summary dataset in terms of sensitivity and specificity. **(b) **In addition, permutation tests carried out using this composite statistic yield *q*-value estimates which are more accurate than any of the 10 component datasets, although still lower than the true false-discovery rate.

We have identified a set of analysis choices that optimally ranks genes according to significance of differential expression. To decide how many of the top genes to investigate further in follow-up experiments, it would be useful to have accurate estimates of the false-discovery rate (FDR or *q*-value), which is the fraction of false positives within a list of genes exceeding a given statistical cutoff. We used our control dataset to compare the actual *q*-values for the 10 optimal expression summary datasets with *q*-value estimates from the permutation method implemented in SAM. As shown in Figure 8b, permutation-based *q*-value calculations using each of the top ten datasets underestimate the actual *q*-value for a given cutoff. We attempted to reduce the contribution of biases inherent in any given data-processing step by combining the results from the top 10 expression summary datasets. The goal is to pinpoint those genes that are called significant regardless of small changes in the analysis protocol (changes that only marginally affect the DEG detection sensitivity and specificity according to our control dataset). To identify these 'robustly significant' genes, we created a combined statistic from the top 10 datasets depicted in Figure 7f, taking into account the significance of each individual test, as well as the variation in fold change between datasets (see Materials and methods). This combined statistic distinguishes between true and false DEGs equally as well as the best of the 10 input datasets (Figure 8a). To make false-discovery rate estimates using this combined statistic, each of the 10 datasets was permuted (using the same permutation) and the combined statistic was recalculated. Figure 8b shows that this combined statistic gives a more accurate *q*-value estimate than any of the individual datasets. However, there is still considerable difference between the estimated and actual *q*-values. For example, if we estimate *q *= 0.05, the corresponding CyberT statistic has an actual *q *= 0.18, and if we estimate *q *= 0.1, then the actual *q *= 0.3. Therefore, until more accurate methods for estimating the false-discovery rate are developed, we recommend that a conservative choice of false-discovery rate cutoff be used (for example < 1%) to prevent actual numbers of false-positive DEG calls (that is, the true, rather than estimated, FDR) from being too high.

### Assessment of sensitivity and specificity

As the identities and relative concentrations of each of the RNAs in the experiment were known, we were able to assess directly the sensitivity and specificity obtained by the best-performing methods. Examination of the ROC curves in Figure 7 reveals that sensitivity begins to plateau as the false discovery rate (*q*) increases from 10% to 30%. Taking an upper acceptable bound for *q *as 10%, the maximum sensitivity obtained is about 71%. Thus, under the best-performing analysis scheme, roughly 380 (29%) of the 1,309 DEGs are not detected as being differentially expressed, with the number of false positives equaling about 105. At *q *= 2%, sensitivity reduces to around 60%, meaning that more than 520 DEGs are missed, albeit with fewer than 20 false positives.

**Figure 9:**
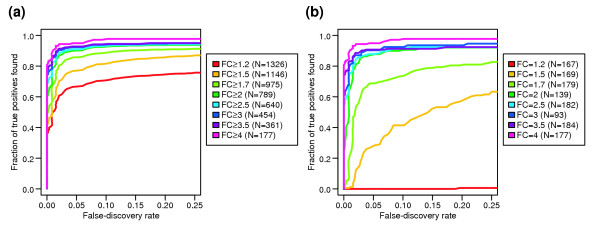
DEG detection sensitivity and specificity as a function of spiked-in fold change level. **(a, b)**ROC curves using the composite statistic, and different definitions of the true-positive probe sets (criteria given in the legends; FC, spiked-in fold change). The true negatives remain the same for all curves (the probe sets which were not spiked in, or were spiked in at 1x).

We next looked at the dependence of sensitivity and specificity on the magnitude of the spiked-in fold-change value. We find that at *q *= 10%, sensitivity is increased to 93% when only cRNAs that differ by twofold or more are considered as DEGs (Figure 9a). This sensitivity decreases only slightly (to 90%) when *q *is lowered to 5%. However, sensitivity drops off sharply as differences in expression below twofold are considered. At *q *= 10%, only 82% of DEGs with 1.5-fold or greater changes in expression are identified, dropping to 71% for all DEGs at 1.2-fold change or above (77% and 67% at *q *= 5%, respectively). The reduction in sensitivity is almost wholly due to the low-fold-change genes: less than 50% of DEGs with fold change 1.5, and none of the DEGs with fold change 1.2, are detected at *q *= 10% (Figure 9b).

It is tempting to conclude from this that we are achieving adequate sensitivity in our experiments and merely need not bother with DEGs below the twofold change level. However, we would argue that obtaining greater sensitivity should be an important goal. There is ample demonstration in the biological and medical literature that small changes in gene expression can have serious phenotypic consequences, as seen both from haploinsufficiencies and from mutations that reduce levels of gene expression through transcriptional regulation or effects on mRNA stability. Furthermore, effective fold changes seen in a microarray experiment might be considerably smaller than actual fold changes within a cell, if the sample contains additional cell populations that dilute the fold-change signal. As it is often not possible to obtain completely homogeneous samples (for example, when profiling an organ composed of several specialized cell types), this is likely to prove a very real limitation to detecting DEGs. In cases where pure cell populations can be obtained, for example by laser capture microdissection, the numbers of cells are often small and RNA needs to undergo amplification in order to have enough for hybridization. Here, non-linearities in RNA amplification might also lead to observed fold changes that fall below the twofold level. We used three microarray replicates for this study, as this is frequently the number chosen by experimentalists because of cost and limiting amounts of RNA. One possible extension of this work would be to examine how many replicates are necessary for reliable detection of DEGs at a given fold change level.

## Conclusions

We have compared a number of popular analysis options for the purpose of identifying differentially expressed genes using an Affymetrix GeneChip control dataset. Clear differences in sensitivity and specificity were observed among the analysis method choices. By trying all possible combinations of options, we could see that choices at some steps of analysis are more critical than at others; for example, the normalization methods that we considered perform similarly, whereas the choice of the PM adjustment method can strongly influence the accuracy of the results. On the basis of our observations, we have chosen a best route for finding DEGs (Figure 3). As any single choice of analysis methods can introduce bias, we have proposed a way to combine the results from several expression summary datasets in order to obtain more accurate FDR estimates. However, these estimates remain substantially lower than actual false-discovery rates, demonstrating the need for continued development of ways to assess the false-discovery rate in experimental datasets. Our analysis further revealed the existence of a high false-negative rate (low sensitivity), especially for those DEGs with a small fold change, and thus suggests the need for improved analysis methods for Affymetrix microarrays. In order to be feasible, this study investigated only a fraction of the current options. The raw data from our hybridizations are available in Additional data files 6-7 and on our websites [27,28], and we encourage the use of this dataset for benchmarking existing and future algorithms. Also important will be the construction of additional control datasets to explore issues not well covered by the present study, such as performance of the analysis methods for specifically detecting low-abundance RNAs and the effects of including larger numbers of replicate arrays. We hope that these experiments will help researchers to choose the most effective analysis routines among those available, as well as guide the design of new methods that maximize the information that can be obtained from expression-profiling data.

## Materials and methods

### cRNA and hybridization

PCR products from *Drosophila *Gene Collection release 1.0 cDNA clones [16] were generated in 96-well format, essentially as described [29]. Each PCR product includes T7 and SP6 promoters located 5' and 3' to the coding region of the cDNA, respectively. Each PCR reaction was checked by gel electrophoresis for a band of detectable intensity and the correct approximate size. Those clones which did not yield PCR product were labeled as 'failed' and eliminated from subsequent analysis. From sequence verification of randomly selected clones, we estimate the number of mislabeled clones to be < 3%. The contents of the plates were collected into 19 pools, such that each pool contained the PCR product from one to four plates (approximately 96-384 clones). Biotinylated cRNA was generated from each pool using SP6 polymerase (detailed protocol available upon request) and the reactions were purified using RNeasy columns (Qiagen). Concentration and purity for each pool was determined both by spectrophotometry and with an Agilent Bioanalyzer. The labeled products were then divided into each of two samples - constant (C) and spike (S) - at specific relative concentrations (Table 1, Figure 1). Because the C sample contains less total RNA than the S sample, 20 μg of (unlabeled) poly(C) RNA was added to the C sample to equalize the nucleic acid concentrations. By mixing the labeled pools just before hybridization, we ensured that the fold change between C and S is uniform for all RNAs within a single pool, while still allowing the absolute concentrations of individual RNAs to vary. The two samples were then hybridized in triplicate to Affymetrix *Drosophila *arrays (DrosGenome1) using standard Affymetrix protocols. We chose to hybridize each replicate chip from an aliquot of a single C (or S) sample, resulting in technical replication; thus this dataset does not address the noise introduced by the labeling and mixing steps. The clones comprising each pool can be found in Additional data file 8, and the resulting Affymetrix chip intensity files (.CEL) files are available in Additional data files 6-7.

### Estimate of RNA concentrations

The total amount of labeled cRNA that was added to each chip (approximately 18 μg) was comparable to a typical Affymetrix experiment (20 μg). Although we do not know the individual RNA concentrations, we estimate that these span the average RNA concentration in a biological GeneChip experiment. Our biological RNA samples typically result in about 40% of the probe sets on the DrosGenome1 chip called present, so the mean amount of individual RNA is 20 μg/(14,010 × 0.40) = 0.003 μg/RNA. In the C chips, the average concentration of individual RNAs in the different pools range from 0.0008 to 0.007 μg/RNA, so the concentrations are roughly similar to those in a typical experiment. We note, however, that there is no way to ensure that the concentration distribution is truly reflective of a real RNA distribution. This is especially true with respect to the low end of the range, as it is usually unknown how many of the absent genes on an array are truly absent versus weakly expressed and thus poorly detected by the analysis algorithms used. Therefore, our analysis possibly favors methods that perform best when applied to highly expressed genes.

### Software

All of the analysis was performed using the statistical program R [30], including the affy and gcrma packages from Bioconductor [18], and scripts adapted from the hdarray library by Baldi *et al*. [31,32]. In addition, we used the dChip [19], MAS 5.0 [12], Perfect Match [20,21] and SAM [27] executables made available by the respective authors. Note that the false-discovery rate calculations were slightly different depending on the *t*-statistic variant: for the SAM statistic, false discovery rates from the authors' Excel Add-in software was used, whereas for the CyberT and basic *t*-statistics, the Bioconductor false-discovery rate implementation was applied, which includes an extra step to enforce monotonicity of the ROC curve. In our experience, this extra step does not qualitatively alter the results. All scripts generated in this study are available for use [27,28].

### Calculation of the statistic that combines the results of multiple expression summary datasets

Say we have *n *datasets and *C*_*ij*_, *S*_*ij *_are the logged signals for a given probe set in the *j*th C and S chips, respectively, in dataset *i*. The mean signal (for this probe set) for the C chips in dataset *i *is:



where  is the number of C chips in dataset *i*; similarly, the mean signal for the S chips in dataset *i *is:



The mean fold change over all datasets is:



The modified standard deviation for the C chips in dataset *i *is based on the CyberT estimate:



where *const *is the weight for the contribution of the average standard deviation  for probe sets with the same average signal intensity as *C*_*ij*_. The modified standard deviation for the S chips in dataset *i *(*sd.S*_*i*_) is defined analogously. The pooled variance over all 10 datasets is defined as:



The variance between the 10 datasets is defined as:



Then the combined statistic was chosen to be:



## Additional data files

Additional data is available with the online version of this article. Additional data file contains a figure and explanatory legend showing the degree of overlap between two lists of differentially expressed genes. Additional data file 2 lists all analysis option combinations used to generate the expression summary datasets in this study. Additional data file 3 is a plot of observed vs actual spiked-in fold changes at the probe level. Additional data file 4 shows an example of asymmetric M (log_2 _fold change) vs A (average log_2 _signal) plot for the comparison of two biological samples. Additional data file 5 contains a comparison of the analysis methods with respect to the detection of DEGs with low signal. Additional data file 6 is a Zip archive containing plain text files (in Affymetrix CEL format), Affymetrix *.CEL files for the C chips in this dataset. Additional data file 7 is a Zip archive containing plain text files (in Affymetrix CEL format), Affymetrix *.CEL files for the S chips in this dataset. Additional data file 8 contains detailed information for the individual DGC clones used in this study.

## Supplementary Material

Additional data file 1 A figure and explanatory legend showing the degree of overlap between two lists of differentially expressed genes Click here for additional data file

Additional data file 2 All analysis option combinations used to generate the expression summary datasets in this study Click here for additional data file

Additional data file 3 A plot of observed vs actual spiked-in fold changes at the probe level Click here for additional data file

Additional data file 4 An example of asymmetric M (log_2 _fold change) vs A (average log_2 _signal) plot for the comparison of two biological samples Click here for additional data file

Additional data file 5 A comparison of the analysis methods with respect to the detection of DEGs with low signal Click here for additional data file

Additional data file 6 A Zip archive containing plain text files (in Affymetrix CEL format), Affymetrix *.CEL files for the C chips in this dataset Click here for additional data file

Additional data file 7 A Zip archive containing plain text files (in Affymetrix CEL format), Affymetrix *.CEL files for the S chips in this dataset Click here for additional data file

Additional data file 8 Detailed information for the individual DGC clones used in this study Click here for additional data file

## Figures and Tables

**Table 1:** The number of clones and assigned fold change for each pool of PCR products

Pool number	Number of clones	Number of assigned Affymetrix probe sets	Assigned fold change (S vs C)	Amount of RNA added to each C chip (μg)	Amount of RNA added to S chip (μg)
1	87	84	1.2	0.47	0.56
2	141	143	2	0.43	0.85
3	85	83	1.5	0.35	0.52
4	180	185	2.5	0.73	1.82
5	90	89	1.2	0.29	0.35
6	88	96	3	0.65	1.94
7	186	188	3.5	0.76	2.67
8	90	95	1.5	0.44	0.67
9	180	190	4	0.78	3.11
10	183	191	1.7	0.48	0.81
13	391	385	1	0.37	0.37
14	369	355	1	1.23	1.23
15	394	404	1	0.40	0.40
16	452	453	1	0.57	0.57
17	419	434	1	0.44	0.44
18	372	407	1	0.31	0.31
19	163	191	1	0.27	0.27

Also depicted is the total amount of cRNA for each pool that was placed on each chip, and the number of Affymetrix probe sets that are assigned to each pool. There were 10,131 probe sets not assigned to any spiked-in clone (called empty). Pools 11 and 12 were not included in this dataset.
