# Supplementary material for: Preferred analysis methods for Affymetrix GeneChips revealed by a wholly defined control dataset
Source: Genome Biol. 2005 Jan 28;6(2):R16. doi: 10.1186/gb-2005-6-2-r16 (PMC551536; doi:10.1186/gb-2005-6-2-r16)
Supplement: Additional data file 1 — A figure and explanatory legend showing the degree of overlap between two lists of differentially expressed genes [file gb-2005-6-2-r16-s1.doc]

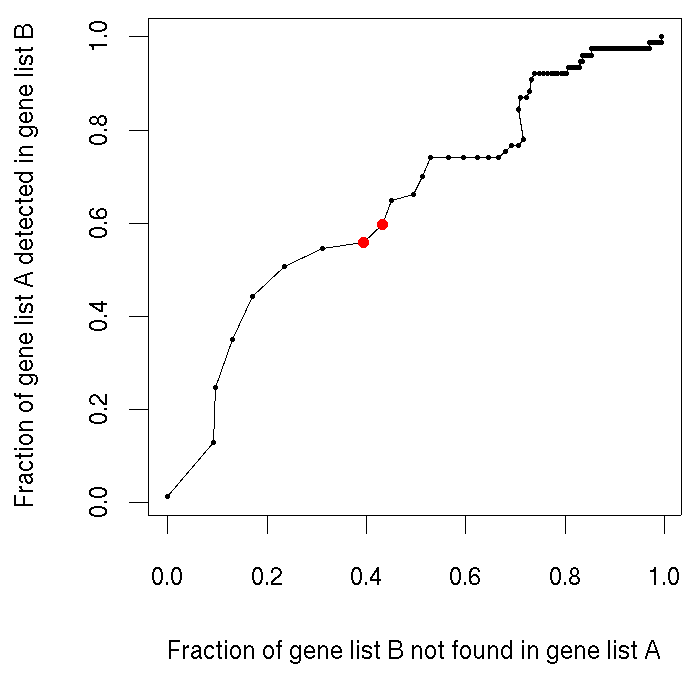


**Additional Data File 1.** The choice of analysis method strongly influences the resulting list of differentially expressed genes. Here we show the degree of overlap between two lists of differentially expressed genes, generated with different analysis methods on the same dataset (a biological experiment comparing genotype *vs.* wild-type, performed in triplicate). Briefly, gene list A involved applying the dChip and RMA software, each followed by SAM, and the results combined using the Fisher method for combining p-values. Gene list B was generated by using the protocol from in this study. Here, gene list A is kept as a static list of genes (using a fold change cutoff = 1.5 and q-value cutoff = 0.05; N=77). Each dot in the figure represents a different gene list B, generated by applying a different q-value cutoff. The location of each dot depicts the degree of overlap between gene list A and gene list B. The red dots correspond to q-value cutoffs where gene list B contains 70 (left dot) and 80 (right dot) genes, showing that when we consider the top 70-80 genes for each method, the overlap between the two lists is only about 60%.
