# Supplementary material for: Preferred analysis methods for Affymetrix GeneChips revealed by a wholly defined control dataset
Source: Genome Biol. 2005 Jan 28;6(2):R16. doi: 10.1186/gb-2005-6-2-r16 (PMC551536; doi:10.1186/gb-2005-6-2-r16)

**Additional Data File 3.** Plot of observed *vs*. actual spiked-in fold changes at the probe level. The control dataset was processed with MAS background correction and quantile normalization before computing fold changes for individual PM probes between the S and C chips. These fold changes are compared to the expected spiked-in fold changes as determined by the probe assignment process described in the Methods. To alleviate potential problems with specific cross-hybridization, only probes that map uniquely to one clone are included in this figure. This boxplot shows that even at the probe level, fold changes levels are lower than the actual values, suggesting that the reduction in fold change magnitude occurs before the probe intensities are combined into expression summary values. The yellow line depicts the result of a simple linear regression, with the parameters shown below the plot.


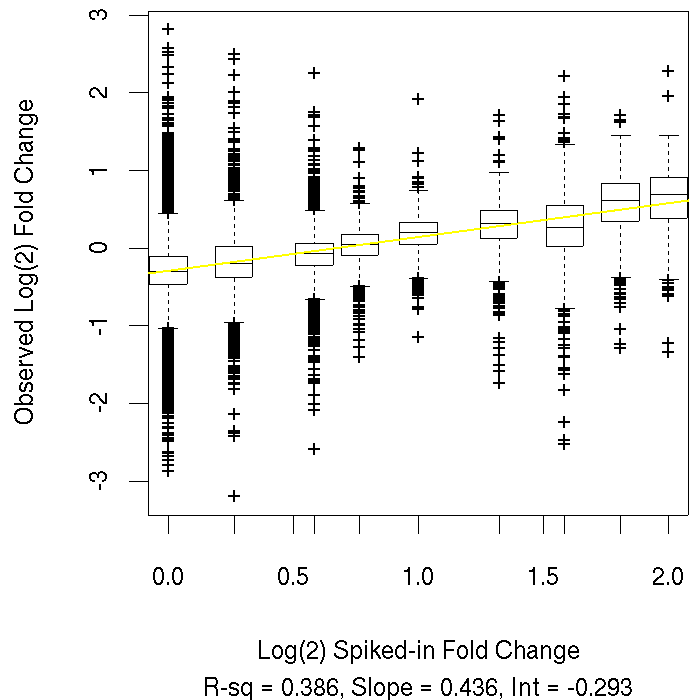

Supplement: Additional data file 3 — A plot of observed vs actual spiked-in fold changes at the probe level [file gb-2005-6-2-r16-s3.doc]
