# Supplementary material for: Preferred analysis methods for Affymetrix GeneChips revealed by a wholly defined control dataset
Source: Genome Biol. 2005 Jan 28;6(2):R16. doi: 10.1186/gb-2005-6-2-r16 (PMC551536; doi:10.1186/gb-2005-6-2-r16)

**Additional Data File 4.** Example of asymmetric M (log_2_ fold change) *vs.* A (average log_2_ signal) plot for the comparison of two biological samples. Although these two chips were processed in parallel, and loess normalization was performed at the individual probe level, we still observe that the M *vs.* A plot is not centered about M = 0 at the probe set level. The orange line is the result of the loess smoothing function on the data. In cases such as this, performing a second normalization step at the probe set level, adjusting the orange line to be closer to the red (M = 0) line, is recommended to avoid spurious DEG calls.


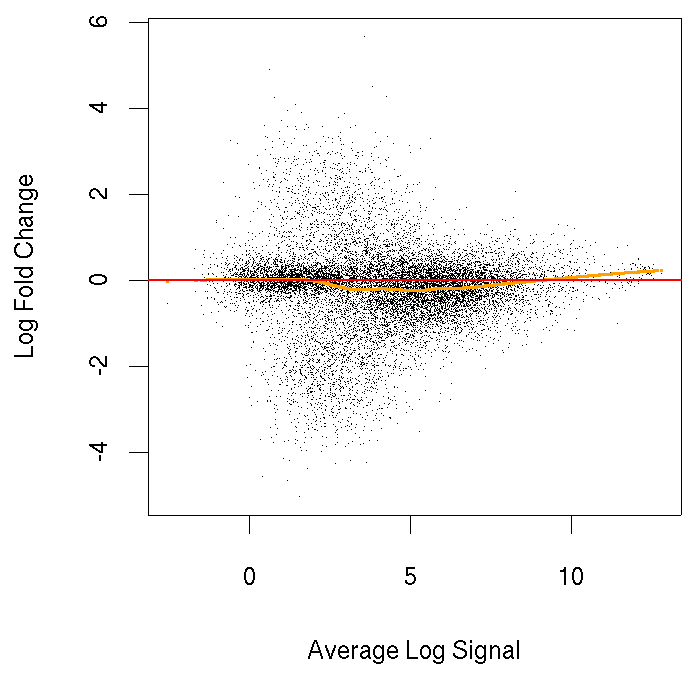

Supplement: Additional data file 4 — An example of asymmetric M (log2 fold change) vs A (average log2 signal) plot for the comparison of two biological samples [file gb-2005-6-2-r16-s4.doc]
