# Supplementary material for: Preferred analysis methods for Affymetrix GeneChips revealed by a wholly defined control dataset
Source: Genome Biol. 2005 Jan 28;6(2):R16. doi: 10.1186/gb-2005-6-2-r16 (PMC551536; doi:10.1186/gb-2005-6-2-r16)
Supplement: Additional data file 5 — A comparison of the analysis methods with respect to the detection of DEGs with low signal [file gb-2005-6-2-r16-s5.doc]

The information in Table 1 indicates that the RNAs spiked in at fold change > 1 have higher average concentration than those at fold change = 1. We sought to better understand the consequence of this fact in the present analysis.

Figure ADF5-1 shows the distribution of expression summaries as a function of spiked-in fold change, for a representative expression summary dataset. Shown as fold change = 0, are probe sets whose target RNA was not spiked in at all (their actual fold change is 1, but they are shown separately from the probe sets which were spiked in). First, we observe that the ranges of summary levels are similar for all fold changes > 1. Therefore, the conclusion from Fig. 9, that smaller fold changes are harder to detect, is not confounded by a possible correlation of lower fold change with lower signal intensity among true positives. Second, we see a clear tendency toward lower signal intensity for the probe sets that were spiked in at fold change = 1.

**Figure ADF5-1**. Boxplots showing the distribution of average log_2_ expression summary intensity (over all S and C chips) as a function of spiked-in fold change. The probe sets that were not spiked in (and thus have fold change = 1) are represented at fold change = 0 in this figure, in order to view them separately from the probe sets which were spiked in at fold change = 1.

It is possible that the low-signal probe sets at fold change = 1 actually represent RNA species which did not amplify due to insufficient starting concentrations. To observe how the expression summary distributions look in the context of the “absent/present” calls from MAS 5.0, the signals were represented as histograms instead of boxplots (Figure ADF5-2), with the histograms for the component “absent” and “present” probe sets superimposed. This figure clearly shows, as expected, that non-spiked in probe sets are overwhelmingly called absent, and spiked-in probe sets are mostly called present. At fold change = 1, however, a small fraction (15%) of the probe sets with fold change = 1 are called “absent”; these have log_2_(signal) = 0 – 6. As these “absent” probe sets closely mirror the distribution for fold change = 0, we hypothesize that these low-signal probe sets represent truly absent RNA. In addition, probe sets with log_2_(signal) = 6 – 10 are not as frequent at fold change > 1 as they are at fold change = 1 (Figure ADF5-3). These probe sets are typically called “present” by MAS 5.0, and thus are possibly targeting low RNA concentrations which are somewhat underrepresented in the set of fold change >1 true positives.

In order to compare the different analysis methods with respect to detecting DEGs at low concentrations, ROC curves were re-calculated, setting as true positives the probe sets with fold change > 1 and signal < 10. There were 265 probe sets that satisfied this criterion, distributed roughly equally among the possible spiked-in fold changes. The true negatives were kept to be the same as before (either not spiked in, or spiked in at fold change = 1). The options at each step of analysis were then compared, analogous to what was done in Fig. 7. Figure ADF5-4 shows that although a smaller fraction of the low-signal DEGs are detected overall, the same options that performed well in Fig. 7 are also optimal here. In brief, the background correction and PM correction methods from MAS 5.0, the median polish and MAS 5.0 expression summary metrics, and loess normalization at the probe set level, are most effective at pinpointing low-signal probe sets. In addition, the gcrma method ranks among the highest-performing (orange lines in most panels of Fig. ADF5-4).

In summary, we posit that the options highlighted in Fig. 3 also perform well at detecting low-signal DEGs.

**Figure ADF5-2**. Histograms of the signal intensity for probe sets spiked in at each fold change level (bold black lines, bin size = 0.2). Superimposed: Frequencies for the subset of probe sets called “present” (thin blue lines) and “absent” (thin red lines) when using the MAS 5.0 absent/present call.

**Figure ADF5-3**. Histograms of the signal intensity for probe sets spiked in at each fold change level (bold black lines, bin size = 0.2). Superimposed: Frequencies for the subset of probe sets called “present” (thin blue lines) and “absent” (thin red lines) when using the MAS 5.0 absent/present call. The top two panels are the same as the analogous plots Fig. ADF5-2, but the third panel is the sum of panels 3-8 from Fig. ADF5-2.

**Figure ADF5-4**. ROC curves for the different analysis methods when using a subset of low-signal spiked-in probe sets as the true positives. See the legend of Fig. 7 for details.
